# Phase II study of pembrolizumab plus nab-paclitaxel in Hormone Receptor–positive, HER2- negative metastatic breast cancer: clinical and molecular insights into response

**DOI:** 10.21203/rs.3.rs-11146137/v1

**Published:** 2026-10-22

**Authors:** Natalie J. Klar, Judith D. Goldberg, Apoorvi Tyagi, Alessandro Leal, Tsivia Hochman, Barb Barbury, Farbod Darvishian, Maryann Kwa, Marleen Meyers, Yelena Novik, Ruth Oratz, Madonna Hanna, Cynthia Loomis, Yury Patskovsky, Douglas K. Marks, Iryna Voloshyna, Michelle Krogsgaard, Sylvia Adams

## Abstract

Purpose We investigate the addition of PD-1 blockade to chemotherapy in HR + MBC to assess efficacy, safety, and changes in the tumor microenvironment utilizing serial tumor biopsies. Methods Single-arm phase 2 clinical trial (NCT02752685) of 20 HR+/HER2- MBC patients received nab-paclitaxel (100mg/m2 IV d1/8, q 3 weeks) and pembrolizumab (200mg IV d1, q 3 weeks, starting with cycle 2). Primary endpoint: best overall response rate (BORR) by RECIST v1.1; secondary endpoints: safety, progression free survival (PFS), duration of response (DOR), and overall survival (OS). Changes in PD-L1 score, stromal tumor-infiltrating lymphocytes (TIL), T-Cell Receptor (TCR) sequence, gene expression, and quantitative multiplex immunofluorescence (qMIF) were evaluated serially during treatment. Results The BORR for partial response (PR) was 26.3% (5/19). Median (m) PFS was 4.1 months (mo) (95% CI 2.2–12.5), mOS was 15.7 mo (95% CI 10.0- NA), and mDOR was 5.6 mo (3.9–52.7). 14 patients experienced one or more grade 3 or higher adverse events (AEs), the most common being anemia, fatigue, diarrhea, and neutropenia, and there were no grade 5 AEs. Longitudinal spatial qMIF and transcriptomic profiling indicated that tumor immune remodeling and proliferative programs shape treatment response, with adaptive immune activation alone being insufficient, supporting combination approaches targeting tumor cell-cycle control and immune competence. No predictive biomarkers emerged, however higher T-cell fraction at baseline was associated with OS (p = 0.0009). Conclusions Chemoimmunotherapy was efficacious with manageable toxicity profile. Importantly, responses were seen in patients previously treated with CDK 4/6 inhibitors. Further research is needed for predictive biomarkers in HR+/HER2- MBC to checkpoint blockade.

